# A retrospective study of histological outcome for IPMN after surgery in Lausanne, Switzerland: A case series

**DOI:** 10.1016/j.amsu.2020.10.028

**Published:** 2020-10-20

**Authors:** Alexis Litchinko, Kosuke Kobayashi, Nermin Halkic

**Affiliations:** a Department of Surgery, Division of Visceral Surgery, University Hospitals of Geneva, Geneva, Switzerland; b Department of Visceral Surgery, Lausanne University Hospital and University of Lausanne, Lausanne, Switzerland

**Keywords:** Intraductal papillary mucinous neoplasm (IPMN), Malignant transformation, Pancreatic cancer

## Abstract

**Introduction:**

Intraductal papillary mucinous neoplasm (IPMN) is a 21st century concept and its management is still controversial. Strong guidelines suggest that surgery is the safest way to prevent malignant evolution. Though the risk of neoplasia is still debated, high-morbidity and mortality surgery must be proposed for high-risk patients to prevent malignant and most likely fatal pancreatic neoplasia.

**Methods:**

The aim of this study was to analyze histological results of patients who underwent operation for IPMN under the Sendai and Fukuoka guidelines. From January 2005 to August 2016, 491 consecutive patients who underwent pancreatic resection in Lausanne University Hospital were analyzed, including 18 IPMN with surgical indication according to the Sendai and Fukuoka criteria.

**Results:**

Thirteen (68.4%) patients had benign histopathology after surgery (the non-malignant group). Of the patients with malignant pathology, four (21%) had high-grade dysplasia and two (20.1%) had invasive carcinoma (the malignant group). The median patient age (*p* = 0.011) and preoperative Carbohydrate Antigen 19–9 (CA19-9) (*p* = 0.030) were significantly higher in the malignant group than in the non-malignant group.

**Discussion:**

The use of the current criteria is adequate, but it may be resulting in surgery on excessive numbers of patients with IPMN. A modern decision-making strategy should be based on clinical features, precise imaging data, and biological markers.

## Highlights

• IPMNs are pre-cancerous tumors with a potential evolution to malignant neoplasm.
• Revised guidelines can lead to surgical decision but with high morbidity and mortality linked to pancreatic surgery.
• Moderns biological markers can help to adjust surgical criteria, added to clinical and imaging features.
• More specific criteria are needed prior to resection, and could lead to more and more conservative management.

## 1 Introduction

Intraductal papillary mucinous neoplasm (IPMN) are rare entities that can be located from the hepatic bile duct (IPMN-Bs) to the pancreatic ducts [1,2]. First described in 1982 [3], then categorized as a different entity from mucinous cystic neoplasms by the lack of ovarian stroma characteristics in 1999. IPMNs are characterized by epithelial proliferation, mucin production that leads to cystic dilatation of the involved ducts, and the potential to evolve to pancreatic malignancy. With the development of several imaging modalities, the prevalence of IPMNs rises up-from 18% to 41% in recent literature, and high correlation with age and diabetes has been reported [[4], [5], [6]]. For many years, the diagnosis and management of these tumors have been strongly influenced first by the Sendai criteria and then by the first version of the Fukuoka guidelines, which were revised in 2017 [7,8]. Previous reports developed criteria and guidelines to evaluate the indications for surgery [9,10]. The clinical parameters used to recommend resection of IPMN are mainly obstructive jaundice, abdominal pain/history of pancreatitis, mural nodule, main duct size, cytology, cyst size, and specific carbohydrate antigen 19–9 (CA 19–9) level. The high risk of evolution to pancreatic adenocarcinoma often leads to pancreatectomy with high risk of morbidity and mortality [11]. Patients are initially evaluated for the presence of high-risk stigmata through endoscopic ultrasound (EUS) or pancreatic juice cytology and, if negative, assessed for other worrisome features, both defined by the Sendai and Fukuoka criteria. The results lead either to surgery or to complementary investigation that aims to precisely evaluate the risk of malignant evolution.

Whether solitary or multiple, those lesions are classified as “main-duct” (MD) or “branch-duct” (BD). According to the guidelines, communication between the cystic lesion and the ductal system is essential for further involvement. There are four subtypes, based on immunohistology. The most common type is the gastric type, which express the mucin proteins 5AC (MUC5AC) and 6 (MUC6) and are mostly of the BD type. The second-most frequent is the intestinal type, mostly found in the pancreatic head, with MD-IPMN expressing MUC2 and MUC5AC. The third is the pancreatobiliary type, expressing MUC1 and MUC5AC and located either in the MD or BD. The fourth and rarest type is oncocytic [12,13], with a <5% prevalence. Each type has a specific intrinsic risk of progression to invasive disease, and these specific risks are still a matter of controversy. A practical algorithm has been reported by Tanaka regarding surveillance criteria and surgical approaches.

Even though the recent 2017 revised guidelines seem to be more conservative with regard to surgical approach, our intent was to show that many IPMN with positive surgical indications are non-malignant tumors by analyzing the histological results of patients who underwent surgical procedures for IPMN that were indicated by the Sendai and Fukuoka guidelines in use at the time of their operations.

## 2 Materials and methods

**Fig. 1:**
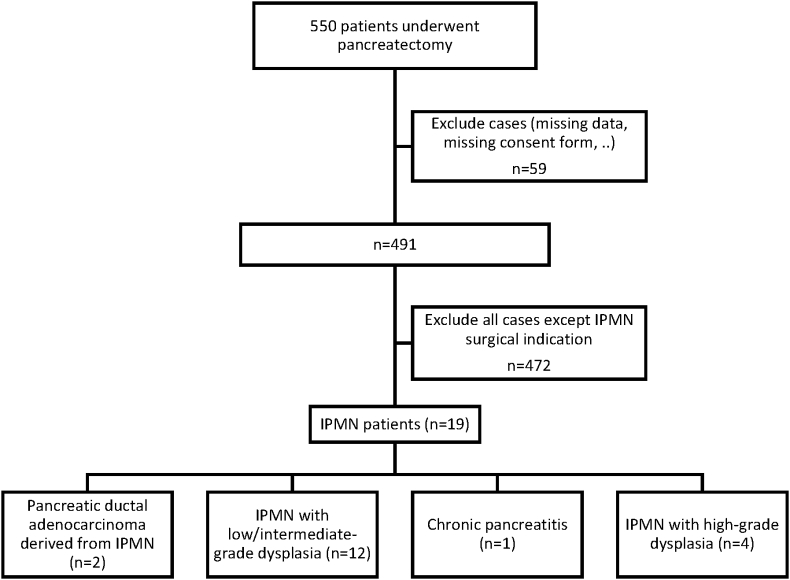
Flowchart of patients who underwent resection for IPMN diagnosis from January 2005 to August 2016.

This study was a retrospective review of 550 consecutive patients who underwent pancreatectomy at the Lausanne University Hospital from January 2005 to August 2016. Of these patients, 19 were diagnosed with IPMN. Of these, 14 underwent pancreaticoduodenectomy, 3 distal pancreatectomy, 1 enucleation, and 1 total pancreatectomy (Fig. 1).

Among these 19 patients, the patient and tumor characteristics of patients who were diagnosed definitively either with high-grade dysplasia or invasive carcinoma (malignant group) were compared with those of the patients who were not diagnosed definitively with malignancy (non-malignant group). All cases were discussed in multidisciplinary meetings and in all the decision to operate was made according to the Sendai and Fukuoka criteria, exploiting all paraclinical exams such as Computed Tomography (CT scan), Magnetic Resonance Imaging (MRI), EUS and laboratory tests. Complications after surgery have been classified with Clavien classification [14] from minor complication (grade I) to death of the patient (grade V).

This manuscript protects patients’ privacy and anonymity. Written informed consent was obtained from all patients and the study was approved by the local ethics committee and registered under the record number Swissethics BASEC 2016-02227 (https://swissethics.ch). This work has been reported in line with the PROCESS 2018 criteria [15].

### 2.1 Statistical analysis

Categorical variables were compared using Fisher's exact test or the chi-square test as appropriate. Continuous variables were compared using the Wilcoxon rank-sum test. A *p* value of <0.05 was considered to indicate statistical significance. The statistical analysis was performed using statistical software (JMP 13.2.0; SAS Institute Inc., Cary, NC, USA).

### 2.2 Pathologic examination

In this retrospective study, the term *malignant* is used as defined in the 2017 guidelines and includes such conditions as invasive carcinoma and high-grade dysplasia. The latter term has been preferred to “carcinoma in situ” and should be abandoned as outlined in the WHO classification [16]. Each histological subtype defined by Furukawa in 2003 has a specific description. There are four distinct subtypes based on morphological and immunohistochemical features: gastric, intestinal, pancreatobiliary, and oncocytic. For example, gastric subtype IPMNs usually have low-grade dysplasia while other subtypes are more likely to develop high-grade dysplasia [7].

## 3 Results

Of the 550 consecutive patients who underwent pancreatectomy in our department, 491 had exploitable data. Of these patients, 19 underwent pancreatectomy for the indication of an IPMN according to the Sendai or Fukuoka criteria. The patients' characteristics and clinical features are summarized in Table 1.

**Table 1:** Patient characteristics and clinical features.

		n = 19
AGE (YEARS)		64 (41–75)
SEX	Male	8 (42.2%)
	Female	11 (57.8%)
BODY MASS INDEX (KG/M^2^)		24.8 (19.2–41.6)
JAUNDICE		2 (10.5%)
APPROACH	Open	19 (100%)
	Laparoscopy	0
ASA PHYSICAL STATUS CLASSIFICATION SYSTEM (ASA)		2 [2,3]
lENGTH OF STAY AFTER SURGERY (DAYS)		19 (4–65)
POST-OPERATIVE COMPLICATIONS (CLAVIEN CLASSIFICATION) [12]	No complications	11 (57.9%)
	I	3 (15.8%)
	II	3 (15.8%)
	III	0
	IV	2 (10.5%)
	V	0
TYPE OF SURGERY	Pancreaticoduodenectomy	14 (73.6%)
	Distal pancreatectomy	3 (15.8%)
	Total pancreatectomy	1 (5.3%)
	Enucleation	1 (5.3%)
DIFFERENTIATION	High-grade	4 (21.0%)
	Moderate	2 (10.5%)
	Low-grade	10 (52.6%)
	Invasive (carcinoma)	2 (10.5%)
	Other (chronic pancreatitis)	1 (5.2%)
HISTOLOGICAL SUBTYPE (n = 18)	Gastric	10 (55.6%)
***Mixte subtypes counted separately***	Oncocytic	0 (0%)
	Intestinal	7 (38.9%)
	Pancreatobiliary	2 (11.1%)
	Mixed	3 (16.7%)
	Unspecified	4 (22.2%)
LABORATORY RESULTS	ASAT (UI/L)	41 (12–170)
	ALAT (UI/L)	52 (10–211)
	Total bilirubin (μmol/L)	40 (32–58)
	Direct bilirubin (μmol/L)	49 (3–255)
	CA 19–9 (U/mL)	112 (2–783)
	CEA (μg/L)	2.2 (0.5–6.1)
TUMOR SIZE (IN MM)	CT/MRI	27.3 (7.0–60.0)
MORPHOLOGY (IN MM)	Main pancreatic duct diameter	7.8 (2.0–18.0)

The median age was 64 years old (range: 41–75), most were female (57.8%), and only 13% had jaundice pre-operation. All surgeries were open approach. Of the 19 patients, 18 were diagnosed histologically with IPMN and only 6 (31.6%) were diagnosed with high-grade dysplasia (21%) or invasive carcinoma (10.5%). One patient with chronic pancreatitis has been excluded from the comparison. The median length of stay after surgery in days was 19 (range: 4–65) and most complications were low grades (<3) according to the Clavien-Dindo classification (89.5%). The histological subtype was gastric in 10 cases (55.6%) and intestinal in 7 (38.9%), 3 patients had mixed subtypes we counted for every category. The subtypes of 4 remaining cases of IPMN (15.0%) were not specified. One patient with MD-type of IPMN who underwent surgery in 2006 was included this study because surgical indication was based on the same criteria as Sendai, such as high-risk stigmata with obstructive jaundice and recurrent cholangitis. The comparison between the two groups, excluding the patient with chronic pancreatitis, is summarized in Table 2. The median patient age and preoperative CA 19–9 were significantly higher in the malignant group than in the non-malignant group (malignant group vs. non-malignant group, age 72 vs. 64 years, *p* = 0.015; and CA 19–9, 137 vs. 5, *p* = 0.030). Although the difference was not significant, the tumors were larger in the malignant group (42.0 mm vs. 29.5 mm, *p* = 0.134).

**Table 2:** Comparison between malignant and non-malignant clinical and paraclinical features.

VARIABLE	MALIGNANT n = 6	NON-MALIGNANT n = 12	*p*-vALUE
AGE, YEAR	72 (63–75)	64 (41–73)	0.015
BODY MASS INDEX (KG/M^2^)	25.4 (16.5–32.4)	24.2 (17.0–41.6)	>0.999
SEX RATIO/MALE: FEMALE	2:4	6:6	0.502
ASA SCORE	2 [2,3]	2 [2,3]	0.668
ASAT (UI/L)	20 (17–95)	23 (12–55)	0.807
ALAT (UI/L)	19 (14–196)	22 (10–108)	0.903
CA19-9 (U/ML)	137 (17–783)	5 (2–57)	0.030
AMYLASE (UI/L)	24 (22–183)	31 (29–59)	0.5676
LIPASE (UI/L)	148 (28–267)	42 (26–112)	0.889
GGT (UI/L)	27 (20–1399)	23 (6–148)	0.3162
ALKALINE PHOSPHATASE (UI/L)	270 (73–467)	70 (48–108)	0.191
TOTAL BILIRUBIN (μMOL/L)	10 (7–258)	10 [[6], [7], [8], [9], [10], [11], [12], [13], [14], [15], [16], [17], [18], [19], [20], [21], [22], [23], [24], [25], [26], [27], [28], [29], [30], [31], [32], [33], [34], [35]]	>0.999
DIRECT BILIRUBIN (μMOL/L)	10 (3–255)	10 [[3], [4], [5], [6], [7], [8], [9], [10]]	0.622
TUMOR SIZE (IRM/CT) (IN MM)	42.0 (24.0–60.0)	29.5 (12.0–45.0)	0.134
TYPE OF IPMN, MAIN/BRANCH/MIXED	2/0/4	2/4/6	0.259
PRE-PATHOLOGICAL EXAMINATION	3 (50.0%)	2 (16.7%)	0.137

There were 5 patients who received a biopsy or preoperative histological examination with EUS, and all histological results were negative for malignancy. Among the 6 patients diagnosed with high-grade dysplasia or invasive carcinoma, 2 had MD-type IPMN, and 4 patients had mixed-type IPMN. For the two invasive carcinoma, pTMN scores were pT1pN0M0R0 and pT3pN1M0R1, with no recurrence for these cases in our institution at the time of our study, after 14 and 9 years of follow-up, respectively. In contrast, among the 12 patients with non-malignant disease, 2 (10.6%) had MD-IPMN, 6 (31.6%) had mixed-type IPMN, and 4 (21.1%) had BD-IPMN. The indications for surgery in the 4 patients with BD-MPN were (i) a lesion over 30 mm in size and increased CA 19–9, (ii) a rapid progression of the tumor size from 11 mm to 36 mm in one year, (iii) a lesion more than 40 mm with worrisome features, (iv) and a 38 mm tumor with mural nodules, respectively.

## 4 Discussion

In this study, we analyzed the histological results and several other parameters of patients who underwent surgical procedures for IPMN. Only 30% of IPMN patients who underwent operation according to the indications set forth in the Sendai and Fukuoka criteria were diagnosed with high-grade dysplasia or invasive carcinoma. The median patient age and preoperative CA 19–9 were significantly higher in the malignant group than in the non-malignant group.

Several studies have shown that about 66% of IPMN patients who underwent operation had no malignancy finding [17,18]. Generally, pancreatectomy has high morbidity and mortality: 35% morbidity and 3% mortality for pancreaticoduodenectomy, 28% and 2% for distal pancreatectomy, and 32% and 5% for total pancreatectomy [18,19]. In fact, 10.5% of patients in this study had a major complication according to the Clavien classification [14], and the median length of hospital stay was 19 days.

Imaging modalities including CT, MRI, and EUS were used according to the recently revised guidelines. Our results suggest that even if the revision of these guidelines has improved the sensitivity of early detection of malignant evolution, further improvement is needed. More than half of our patients had surgery for a low- or intermediate-grade dysplasia. The current revised Fukuoka guidelines for the management of IPMNs have greatly improved the management of these lesions, but this study suggest that their diagnostic sensitivity and specificity must be enhanced. Some studies have shown a sensitive high rate of malignancy in Sendai-negative lesions [20]. More rigorous criteria for surgical decision-making are needed to select patients for operation and to detect those eligible for surveillance but with a considerable risk of malignant transformation.

Imaging by EUS is now an effective means of investigating pancreatic cystic lesions, and other centers recommend using it with fine-needle–guided aspiration in order to search for genetic mutations. In the present study, EUS was performed on 26% of the patients. Recent studies have proposed combining molecular with EUS data for pre-therapeutic cytopathologic testing by searching for KRAS and GNAS mutations in cystic fluid sampled using fine-needle aspiration [[21], [22], [23], [24]]. Wang et al. and Fritz et al. [25,26] showed that pre-operative assessment of serum carcinoembryonic antigen and CA19-9 to identify malignant and invasive IPMNs can also greatly improve the decision to perform surgery. In our study, CA 19–9 was significantly higher in the malignant than in the non-malignant group (*p* = 0.030). Otsakua et al., in a retrospective study [27], recently showed the importance of managing GNAS in identifying high-risk IPMN with concomitant adenocarcinoma. Some authors have suggested using pancreatic juice cytology to enhance preoperative diagnosis by improving the risk classification for malignant IPMN [28]. Another matter of concern is the extent of resection in case of positive margins for high-grade dysplasia or invasive carcinoma. Re-resection to negative margins should be performed even if this requires total pancreatectomy.

Histologic and cyst fluid biomarkers for high-risk IPMN, including KRAS, GNAS, and MUC1/MUC2/MUC4/MUC5A, will be used in future decision making about treatment. MUC1 is known to be associated with a higher risk of invasive carcinoma [29,30], as is MUC4 [31]. Lim et al. [32] developed an interesting list of modern criteria in their review of the recent literature and precisely summarized changes in clinical thinking about IPMN over time. More recently, some authors have suggested that the neutrophil-to-lymphocyte ratio is a good predictive factor of malignancy in IPMN tumors [[33], [34], [35], [36]]. This, too, could be easily measured using preoperative blood samples.

Some previous studies have also questioned the accuracy of the Sendai and Fukuoka criteria. Heckler et al. [37] suggested that the Fukuoka criteria have highly improved sensitivity for BD-IPMN but that either some other pre-operative paraclinical exams are needed to improve their specificity or the decision should be made based on recent recommendations and personal application of recent paraclinical testing [20]. In either case, the decision should stay in the hands of the surgeon and patient and be guided by strong recommendations and clear prognostic involvement. Recent recommendations adopt a more conservative attitude concerning surgical indications for IPMNs. The 2017 revision of the International Association of Pancreatology guidelines by Tanaka et al. [8] and the 2018 revision of the EURO guidelines [38] to a more conservative approach both suggest that our current thinking about IPMNs is still perfectible and that we need other criteria to better evaluate the malignant risk of these tumors. Some surgical teams have proposed an interesting decision tree regarding a specific Asian population [[39], [40], [41]], and in 2019 Tanaka et al. summarized all nine actual recommendations published in the recent English literature [42]. The last five years, many recommendations gave specific resection criteria, surgical indications, surveillance intervals and modalities for non-suspicious IPMN [8,38,[43], [44], [45]]. It appears that the decision making between surgical approach or surveillance is still a matter of debate and the endpoint of this reflection is the balance between patient fitness, moderns imaging system, endoscopic ultrasound, tumor markers with cytology and immunohistochemical analysis of samples. In 2019, WHO classification has been revised in the 5th edition of the WHO Classification of Tumors [46], proposed a precision by grading IPMNs as low grade (previously low and intermediate grades) and high grade (previously high-grade dysplasia and carcinoma in situ). By removing the intermediate grade, this may increase the number of low-grade tumors and lower the number of patient candidate to surgery.

Our study has several limitations. First, it is retrospective. Second, only a small number of patients with IPMN were treated in our institution and we had especially few patients with branch-type IPMN. Histological preoperative examination was performed for only 27.8% of the patients. This study also did not include patients diagnosed with IPMN who had not undergone surgery during the study period. In fact, it is preferable to add the number of developed cancers in the observed group, but it was difficult to find those data. Third, in our institution we were unable to investigate several prognostic factors, such as MUC1.

To conclude, decision-making based on current criteria is almost appropriate, but it may include result in excessive surgery on patients with IPMN. Similar with previous reports, elderly patients and high level of CA19-9 were predictive factors of malignant IPMN. A modern strategy should be based on clinical features, precise imaging data, and biological markers. Multidisciplinary teams should use this information to develop an individualized treatment for each patient, considering comorbidities and life expectancy.

## Ethical approval

Written informed consent was obtained from all patients and the study was approved by the local ethics committee (CER-VD) and registered under the record number 2016-02227.

## Funding

None.

## Author contribution

All authors were involved in study conceptualization and design. Alexis Litchinko collected the data. Alexis Litchinko & Kosuke Kobayashi analyzed and interpreted the data. All authors (Alexis Litchinko; Kosuke Kobayashi & Nermin Halkic) contributed to the writing and editing of the final draft.

## Registration of research studies

Name of the registry: BASEC = Business Administration System for Ethics Committees

Unique Identifying number or registration ID: 2016-02227

Hyperlink to your specific registration (must be publicly accessible and will be checked): https://swissethics.ch/en/basec

## Guarantor

Prof. Nermin Halkic

## Type of study

Case series.

SwissEthics Identifying number ID: BASEC/2016-02227.

## Provenance and peer review

Not commissioned, externally peer reviewed.

## Declaration of competing interest

No financial disclosure or competing interests.

## References

[1] Taouli B., Vilgrain V., O'Toole D. (2002). Intraductal papillary mucinous tumors of the pancreas: features with multimodality imaging. J. Comput. Assist. Tomogr..

[2] Clores M.J., Thosani A., Buscaglia J.M. (2014). Multidisciplinary diagnostic and therapeutic approaches to pancreatic cystic lesions. J. Multidiscip. Healthc..

[3] Ohashi K., Murakami Y., Takeoshi T., Ohta H., Ohashi I. (1982). Four cases of mucin producing cancer of the pancreas on specific findings of the papilla of Vater (Japanese). Prog Dig Endosc.

[4] Aronsson L., Andersson R., Ansari D. (2017). Intraductal papillary mucinous neoplasm of the pancreas - epidemiology, risk factors, diagnosis, and management. Scand. J. Gastroenterol..

[5] Kosmahl M., Pauser U., Peters K. (2004). Cystic neoplasms of the pancreas and tumor-like lesions with cystic features: a review of 418 cases and a classification proposal. Virchows Arch..

[6] Yoon W.J., Lee J.K., Lee K.H. (2008). Cystic neoplasms of the exocrine pancreas: an update of a nationwide survey in Korea. Pancreas.

[7] Tanaka M., Fernández-del castillo C., Adsay V. (2012). International consensus guidelines 2012 for the management of IPMN and MCN of the pancreas. Pancreatology.

[8] Tanaka M., Fernández-del castillo C., Kamisawa T. (2017). Revisions of international consensus Fukuoka guidelines for the management of IPMN of the pancreas. Pancreatology.

[9] Del Chiaro M., Verbeke C., Salvia R. (2013). European experts consensus statement on cystic tumours of the pancreas. Dig. Liver Dis..

[10] Vege S.S., Ziring B., Jain R., Moayyedi P. (2015). American gastroenterological association institute guideline on the diagnosis and management of asymptomatic neoplastic pancreatic cysts. Gastroenterology.

[11] Ho C.K., Kleeff J., Friess H., Büchler M.W. (2005). Complications of pancreatic surgery. HPB.

[12] Hibi Y., Fukushima N., Tsuchida A. (2007). Pancreatic juice cytology and subclassification of intraductal papillary mucinous neoplasms of the pancreas. Pancreas.

[13] Furukawa T., Klöppel G., Volkan adsay N. (2005). Classification of types of intraductal papillary-mucinous neoplasm of the pancreas: a consensus study. Virchows Arch..

[14] Dindo D., Demartines N., Clavien P.A. (2004). Classification of surgical complications: a new proposal with evaluation in a cohort of 6336 patients and results of a survey. Ann. Surg..

[15] Agha R.A., Borrelli M.R., Farwana R. (2018). The PROCESS 2018 statement: updating consensus preferred reporting of CasE series in surgery (PROCESS) guidelines. Int. J. Surg..

[16] Adsay N.V., Fukushima N., Furukawa T., Hruban R.H., Klimstra D.S., Kloppel G., Bosman F.T., Carneiro F., Hruban R.H., Theise N.D. (2010). Intraductal neoplasm of the pancreas. WHO Classification of Tumors of Digestive System.

[17] Heckler M., Michalski C.W., Schaefle S., Kaiser J., Büchler M.W., Hackert T. (2017). The Sendai and Fukuoka consensus criteria for the management of branch duct IPMN - a meta-analysis on their accuracy. Pancreatology.

[18] Venkat R., Puhan M.A., Schulick R.D., Cameron J.L., Eckhauser F.E., Choti M.A. (2011). Predicting the risk of perioperative mortality in patients undergoing pancreaticoduodenectomy: a novel scoring system. Arch. Surg..

[19] Ho C.K., Kleeff J., Friess H., Buchler M.W. (2005). Complications of pancreatic surgery. HPB.

[20] Strobel O., Büchler M.W. (2017). [A nomogram for the prediction of malignancy in branch-duct IPMN]. Chirurg.

[21] Bournet B., Vignolle-vidoni A., Grand D. (2016). Endoscopic ultrasound-guided fine-needle aspiration plus KRAS and GNAS mutation in malignant intraductal papillary mucinous neoplasm of the pancreas. Endosc. Int. Open.

[22] Nissim S., Idos G.E., Wu B. (2012). Genetic markers of malignant transformation in intraductal papillarymucinous neoplasm of the pancreas: ameta-analysis. Pancreas.

[23] Distler M., Aust D., Weitz J. (2014). Precursor lesions for sporadic pancreatic cancer: PanIN, IPMN, and MCN. BioMed Res. Int..

[24] Basturk O., Tan M., Bhanot U. (2016). The oncocytic subtype is genetically distinct from other pancreatic intraductal papillarymucinous neoplasm subtypes. Mod. Pathol..

[25] Wang W., Zhang L., Chen L. (2015). Serum carcinoembryonic antigen and carbohydrate antigen 19-9 for prediction of malignancy and invasiveness in intraductal papillary mucinous neoplasms of the pancreas: a meta-analysis. Biomed Rep.

[26] Fritz S., Hackert T., Hinz U., Hartwig W., Büchler M.W., Werner J. (2011 Jan). Role of serum carbohydrate antigen 19-9 and carcinoembryonic antigen in distinguishing between benign and invasive intraductal papillary mucinous neoplasm of the pancreas. Br. J. Surg..

[27] Ohtsuka T., Tomosugi T., Kimura R. (2019). Clinical assessment of the GNAS mutation status in patients with intraductal papillary mucinous neoplasm of the pancreas. Surg. Today.

[28] Yamakawa K., Masuda A., Nakagawa T. (2019). Evaluation of efficacy of pancreatic juice cytology for risk classification according to international consensus guidelines in patients with intraductal papillary mucinous neoplasm; a retrospective study. Pancreatology.

[29] Furukawa T., Kloppel G., Volkan Adsay N. (2005). Classification of types of intraductal papillary-mucinous neoplasm of the pancreas: a consensus study. Virchows Archi Int J Pathol.

[30] Furukawa T., Hatori T., Fujita I. (2011). Prognostic relevance of morphological types of intraductal papillary mucinous neoplasms of the pancreas. Gut.

[31] Maker A.V., Katabi N., Gonen M. (2011). Pancreatic cyst fluid and serum mucin levels predict dysplasia in intraductal papillary mucinous neoplasms of the pancreas. Ann. Surg Oncol..

[32] Lim J., Allen P.J. (2019). The diagnosis and management of intraductal papillary mucinous neoplasms of the pancreas: has progress been made ?. Updates Surg.

[33] Mcintyre C.A., Pulvirenti A., Lawrence S.A. (2019). Neutrophil-to-Lymphocyte ratio as a predictor of invasive carcinoma in patients with intraductal papillary mucinous neoplasms of the pancreas. Pancreas.

[34] Ohno R., Kawamoto R., Kanamoto M. (2019). Neutrophil to lymphocyte ratio is a predictive factor of malignant potential for intraductal papillary mucinous neoplasms of the pancreas. Biomark. Insights.

[35] Arima K., Okabe H., Hashimoto D. (2015). The neutrophil-to-lymphocyte ratio predicts malignant potential in intraductal papillary mucinous neoplasms. J. Gastrointest. Surg..

[36] Gemenetzis G., Bagante F., Griffin J.F. (2017). Neutrophil-to-lymphocyte ratio is a predictive marker for invasive malignancy in intraductal papillary mucinous neoplasms of the pancreas. Ann. Surg..

[37] Heckler M., Michalski C.W., Schaefle S., Kaiser J., Büchler M.W., Hackert T. (2017). The Sendai and Fukuoka consensus criteria for the management of branch duct IPMN - a meta-analysis on their accuracy. Pancreatology.

[38] European Study Group on Cystic Tumours of the P (2018). European evidence-based guidelines on pancreatic cystic neoplasms. Gut.

[39] Correa-Gallego C., Do R., Lafemina J. (2013). Predicting dysplasia and invasive carcinoma in intraductal papillary mucinous neoplasms of the pancreas: development of a preoperative nomogram. Ann. Surg Oncol..

[40] Jang J.Y., Park T., Lee S. (2016). Proposed nomogram predicting the individual risk of malignancy in the patients with branch duct type intraductal papillary mucinous neoplasms of the pancreas. Ann. Surg..

[41] Shimizu Y., Yamaue H., Maguchi H. (2015). Validation of a nomogram for predicting the probability of carcinoma in patients with intraductal papillary mucinous neoplasm in 180 pancreatic resection patients at 3 high-volume centers. Pancreas.

[42] Tanaka M. (2019). Clinical management and surgical decision-making of IPMN of the pancreas. Methods Mol. Biol..

[43] Aunan J.R., Jamieson N.B., Søreide K. (2019). Observation or resection of pancreatic intraductal papillary mucinous neoplasm: an ongoing tug of war. World J. Gastrointest. Oncol..

[44] Vege S.S., Ziring B., Jain R., Moayyedi P. (2015). Clinical Guidelines Committee; American Gastroenterology Association. American gastroenterological association institute guideline on the diagnosis and management of asymptomatic neoplastic pancreatic cysts. Gastroenterology.

[45] Elta G.H., Enestvedt B.K., Sauer B.G., Lennon A.M. (2018). ACG clinical guideline: diagnosis and management of pancreatic cysts. Am. J. Gastroenterol..

[46] Nagtegaal I.D., Odze R.D., Klimstra D. (2020). The 2019 WHO classification of tumours of the digestive system. Histopathology.

